# Epithelial downgrowth masquerading as granulomatous anterior and intermediate uveitis with histopathologic evidence of 5-FU treatment

**DOI:** 10.3205/oc000245

**Published:** 2024-09-23

**Authors:** Colin P. Froines, Alexander D. Lin, Kaivon Pakzad-Vaezi, Gordana Juric-Sekhar, Caitlin S. Latimer, Kathryn P. Scherpelz, C. Dirk Keene, Eissa M. Hanna, Michael R. Banitt, Luis F. Gonzalez-Cuyar

**Affiliations:** 1 Department of Ophthalmology, University of Washington, Seattle, Washington, USA; 2 Department of Ophthalmology, Kaiser Hawaii, Honolulu, Hawaii, USA; 3 Department of Ophthalmology, University of British Columbia, Vancouver, British Columbia, Canada; 4 Department of Laboratory Medicine and Pathology, Division of Neuropathology, University of Washington, Seattle, Washington, USA; 5 Department of Ophthalmology, Wellish Vision Institute, Las Vegas, Nevada, USA; 6 Department of Ophthalmology, Northwest Eye Surgeons, Arlington, Washington, USA

**Keywords:** epithelial downgrowth, uveitis, masquerade, histopathologic correlation, 5-FU

## Abstract

**Purpose:**

Highlight an unusual case of epithelial downgrowth (EDG) masquerading as granulomatous anterior and intermediate uveitis with histopathologic evidence of 5-fluorouracil (5-FU) treatment.

**Case description:**

A 33-year-old man presented after multiple corneal surgeries and neodymium-doped yttrium aluminum garnet (Nd:YAG) capsulotomies with subacute angle closure, pain, light sensitivity, and decreased vision. Exam was notable for granulomatous keratic precipitates, an opacified lens capsule, and vitreous cell/haze. An anterior chamber paracentesis was sent for 16 s (pan-bacterial) and 28 s (pan-fungal) rRNA polymerase chain reaction testing, which returned negative. Diagnostic argon laser photocoagulation was performed on the iris and lens capsule, which blanched upon laser photocoagulation, and subsequent iris biopsy confirmed the presence of epithelial downgrowth (EDG). The patient was treated with multiple injections of 5-FU with repeat biopsy demonstrating both a reduction and apparent resolution in epithelial cell burden after 5-FU.

**Conclusion:**

This case demonstrates an unusual presentation of EDG in a young patient with granulomatous anterior and intermediate uveitis, where simple office-based procedures of Argon laser photocoagulation and anterior chamber paracentesis helped aid in diagnosis and management. Histopathological examination in serial specimens demonstrated the effect of 5-FU on EGD. To our knowledge, this case is the first to describe histopathological reduction in epithelial cell burden with sustained resolution.

## Introduction

Epithelial downgrowth (EDG) is a rare complication of intraocular surgery. While classic examination findings have been previously described [1], presentation may be variable including iris masses, hypopyon, and anterior uveitis [2], [3]. Given the broad range of presentation, a comprehensive differential diagnosis must first be formulated and excluded before a diagnosis of EDG is made. Work-up requires a high degree of suspicion for vision-threatening etiologies and prompt intervention. 

We describe a case of suspected late onset endophthalmitis following multiple ocular procedures in the context of prominent granulomatous uveitis. Our approach to the differential diagnosis, workup, treatment, and use of histopathologic correlation is discussed.

## Case description

The patient was a 33-year-old man with ocular history of bilateral laser in-situ keratomileusis (LASIK), and post-operative keratectasia (greatest in the left eye), who was status post two left eye full-thickness penetrating keratoplasties (PKP) on presentation to our clinic. After the initial PKP a left eye iris cyst was noted. The subsequent left PKP was combined with cataract extraction with 3-piece intraocular lens implantation into the ciliary sulcus, and biopsy of the iris cyst. Outside histopathology at that time revealed normal iris tissue. Following his second combined left PKP procedure, he developed a membrane over the anterior and posterior surfaces of the implanted lens. Two neodymium-doped yttrium aluminum garnet (Nd:YAG) laser capsulotomies were performed in an attempt to clear the membrane. Following his Nd-YAG capsulotomies he developed subacute angle closure requiring maximum medical therapy, including oral acetazolamide. Despite maximal medical therapy the patient continued to have increased intraocular pressures (IOP) of 38 mmHg in the left eye requiring two anterior chamber paracenteses and was referred to our clinic for further management. 

On presentation to our clinic, the patient was asymptomatic apart from occasional pain during IOP elevations and denied current eye pain, photophobia, fevers, chills, or eye redness. Uveitis review of symptoms was negative except for rare labial cold sores and a history of scratches from cats several years prior. Past medical and family histories were unremarkable, and he was not taking any systemic medications. The patient was found to have a corrected visual acuity by Snellen testing of 20/30 (right eye), 20/500 (left eye) that improved to 20/20 and 20/100, respectively, on pinhole testing. IOP by applanation were 12 mmHg and 21 mmHg for the right and left eyes, respectively. He was noted to have an irregular non-reactive 5 mm mid-dilated pupil in the left eye without afferent pupillary defect by reverse testing. Slit lamp biomicroscopy was within normal limits except for normal appearing LASIK scars for the right eye without any evidence of intraocular inflammation. The left eye was notable for 1+ diffuse conjunctival injection, many large granulomatous-appearing keratic precipitates diffusely with an intact corneal graft without stromal edema and no infiltrates. Anterior chamber exam revealed grade 2+ cell by Standardization of Uveitis Nomenclature (SUN) guidelines, and 1+ flare without hypopyon. There was a large superior iridectomy, consistent with previous iris biopsy, with an iris polypropylene suture in place as well as a posterior chamber intraocular lens with multiple thin deposits on the anterior surface of the lens. A more diffuse fibrotic membrane was noted posterior to the intraocular lens along with vitreous cell and 2+ vitreous haze by indirect ophthalmoscopy. No white infiltrates or plaques were noted on the lens, haptics, or capsule. Gonioscopy was not possible due to the corneal opacity from the large keratic precipitates. Fundus exam was within normal limits for both eyes without evidence of retinitis, vasculitis, or optic nerve edema.

Due to the history of multiple Nd-YAG capsulotomy attempts and cataract/corneal surgery with anterior and intermediate granulomatous uveitis, concern was raised for chronic endophthalmitis. An anterior chamber paracentesis was performed, and a 0.3 mL sample was sent for 16 s (pan-bacterial) and 28 s (pan-fungal) rRNA polymerase chain reaction (PCR) testing. Intravitreal vancomycin was administered (1 mg/0.1 mL) and blood was drawn for syphilis, toxoplasma, QuantiFERON Gold, HLA-B27, complete blood count, and complete metabolic panel in addition to chest radiographs to evaluate for sarcoidosis. The PCR testing, blood work, and chest imaging all returned negative. Diagnostic Argon laser photocoagulation was performed to the surface of the iris, which was suggestive of epithelial downgrowth. 

**Figure 1:**
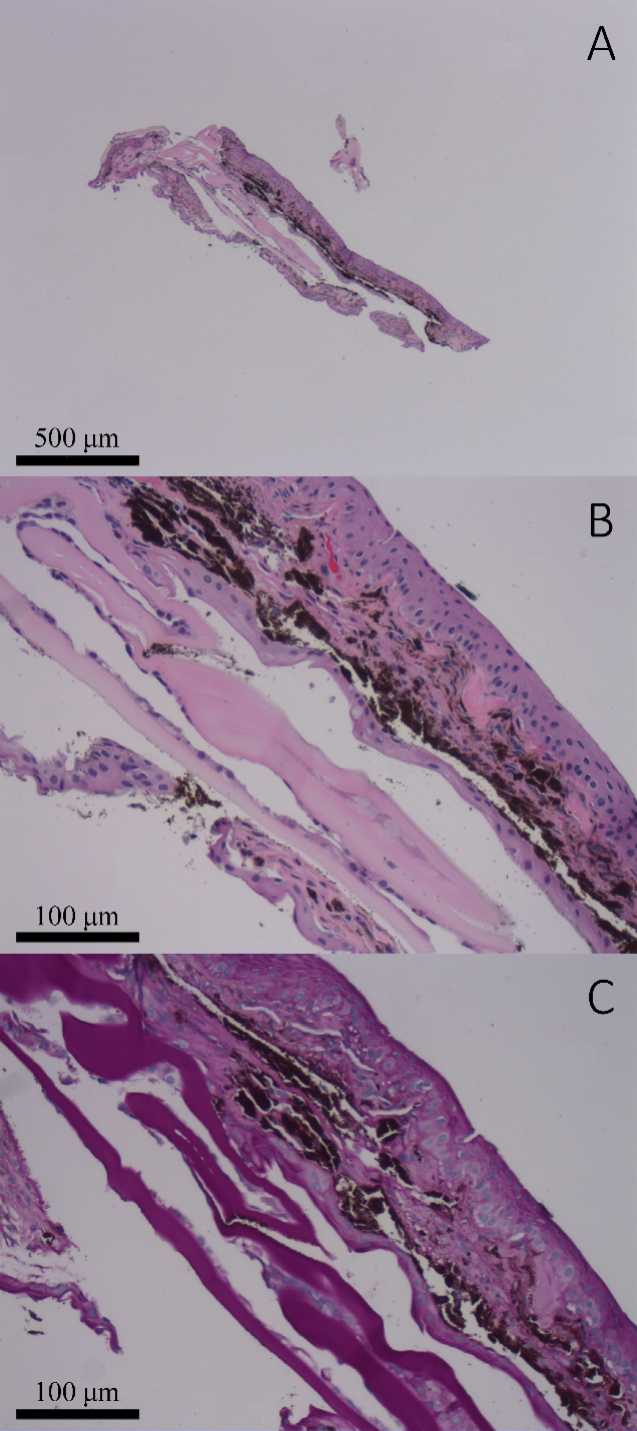
(A–B) Iris biopsy no. 1: Hematoxylin and eosin (H&E) stained sections demonstrate minute detached fragments of lens capsule attached to a portion of uveal tissue which is extensively surfaced by multiple layers of corneal epithelium (4x and 20x, respectively). (C) Periodic acid Schiff (PAS) stained sections highlighted the PAS-positive lens capsule (20x).

The patient required urgent glaucoma surgery following work up for inflammatory and infectious causes returned negative. He underwent a pars plana vitrectomy with Ahmed (New World Medical, Rancho Cucamonga, CA, USA) glaucoma drainage device implantation. The tube insertion site was into the vitreous cavity. At the same time a small piece of iris tissue was removed through two paracentesis incisions using intraocular scissors and forceps and sent for pathology evaluation (Figure 1 (Fig. 1)). At the conclusion of the procedure 0.5 mg in 0.1 mL 5-fluorouracil (5-FU) was injected into the anterior chamber. An undiluted vitreous sample was sent for flow cytometry, surface marker analysis, and PCR for pan-bacterial, pan-fungal, herpes simplex virus (HSV), varicella zoster virus (VZV), cytomegalovirus (CMV), Epstein-Barr virus (EBV), human herpesvirus 8 (HHV8), mycobacterium tuberculosis, and atypical mycobacterial species. The PCR testing returned negative and flow cytometry excluded lymphoma (B-lymphocytes were virtually absent, and the ratio of CD4:CD8 T-lymphocytes was 17:1). 

**Figure 2:**
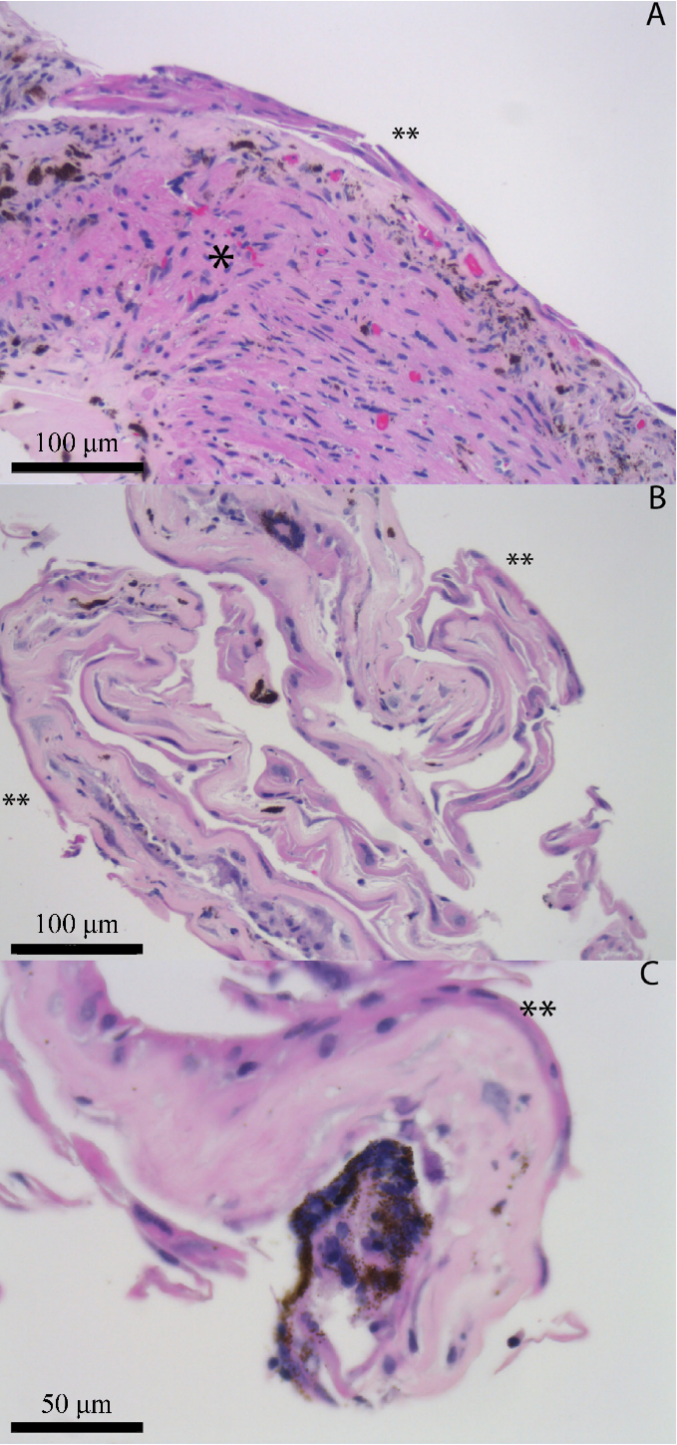
Iris biopsy no. 2: H&E-stained sections demonstrate fragments of iris (denoted by *) and fibrous stroma with associated basement membrane that is partially surfaced by corneal epithelium (denoted by **; generally 1–2 layers thick) (A and B: 20x; C: 40x).

Three weeks post-operatively, the patient began receiving weekly intraocular injections of 0.5 mg in 0.1 mL of 5-FU for a subsequent four weeks. The patient’s intraocular pressure remained stable for one month however increasingly required medical therapy to maintain normal IOP. Immediate postoperative corrected visual acuity was hand motion, which improved to 20/60 at one month. By three months after surgery the patient had a relatively quiet anterior chamber reaction but the granulomatous keratic precipitates changed from discrete spots to a more diffuse pigment clumping throughout the endothelium. At this time his IOP remained elevated to 24–28 mmHg while on maximum tolerated medical therapy including oral acetazolamide. Additionally, his corneal thickness increased to 760 µm at this time with hand motion visual acuity. Decision was made to return to the operating room and perform a third PKP and place a second glaucoma drainage device using Baerveldt (Advanced Medical Optics, Inc., Santa Ana, CA, USA). Pathological examination from the corneal button revealed bullous keratopathy with epithelial attenuation. Additionally, the patient also had an avascular membrane across the intraocular lens that was removed along with a small biopsy of his iris including the pupil border. While the first biopsy demonstrated fragments of iris partially surfaced by an epithelial lining, which was several cell layers thick (Figure 1 (Fig. 1)), the second biopsy after five intraocular injections of 5-FU revealed a portion of iris partially surfaced by an atrophic appearing epithelium which was only 1–2 cells thick (Figure 2 (Fig. 2)).

Post-operatively the patient received intraocular 5-FU injections at weeks two, four, and six. The patient’s visual acuity was counting fingers and anterior chamber remained relatively quiet. At two months post-op the patient developed left eye hypotony with development of appositional choroidals seen on B scan requiring stepwise surgical closure of Ahmed glaucoma drainage device and drainage of choroidals followed by surgical closure of Baerveldt glaucoma drainage device. Subsequently the patient developed significant band keratopathy in the left eye requiring EDTA chelation therapy. Visual acuity with correction pre-chelation was 20/300. Following the development of band keratopathy IOP measurements were unreliable centrally, however IOP was noted to be elevated on repeated measurements taken in 4 quadrants at limbus. The patient underwent laser suture lysis of Baerveldt glaucoma implant with probable IOP lowering as measured at limbus. The patient ultimately underwent combined PKP, anterior vitrectomy, removal of pupillary membrane, removal of intraocular lens with insertion of posterior chamber scleral fixed IOL and revision of glaucoma device. Pathologic evaluation did not identify epithelial downgrowth. 

Following routine post-operative care, with final corrected visual acuity of 20/125 and stably quiet anterior chamber, the patient sought continued care at an outside facility. Months later the patient had a fifth left eye PKP. Pathologic evaluation at this medical center did not identify epithelial downgrowth. 

## Discussion

This case highlights an unusual presentation of EDG occurring with large, mutton-fat granulomatous anterior and intermediate uveitis. This condition occurs when epithelial cells from the cornea or conjunctiva migrate through a wound and proliferate along the intraocular structures. It typically presents either as a diffuse sheet or as multiple cysts within the anterior chamber, the former being more aggressive and difficult to treat [4]. However, a uveitic presentation is atypical. Predisposing conditions for EDG include multiple intraocular surgeries, vitreous in the wound, incomplete or delayed wound healing, as well as suture track leaks [4]. EDG is rare and has been estimated to occur in 0.08–0.12% of cataract surgeries [4], 1.3% of LASIK performed with microkeratome [5], and 0.25% of aphakic penetrating keratoplasties [6]. Given the patient’s distant history of LASIK, it is suspected that EDG occurred following initial PKP. Our patient developed recurrent sheet-like membranes that formed over the lens implant but had additional features of granulomatous anterior and intermediate uveitis raising the suspicion for delayed post-operative endophthalmitis. 

Endophthalmitis must be excluded if an infectious cause of inflammation following surgery is suspected, and an anterior chamber or vitreous paracentesis can be safely performed in clinic [7]. It has been shown that the use of PCR to detect microorganisms in delayed endophthalmitis following cataract surgery is more sensitive than direct microscopy or diagnostic culture [8]. This technique is especially important in cases of suspected *Propionibacterium acnes* or fungal infection, or with previous intravitreal antibiotics, as culture growth can be difficult in these situations [9].

Other diagnostic considerations that should be prompted by this patient’s presentation include sympathetic ophthalmia, mechanical irritation (such as uveitis-glaucoma-hyphema [UGH] syndrome), infectious causes of granulomatous inflammation, such as syphilis, tuberculosis, or the human herpes virus family, and non-infectious causes, such as sarcoidosis. The lack of bilateral inflammation effectively ruled out sympathetic ophthalmia. Although the intraocular lens was placed in the sulcus, it was well positioned, was a 3-piece lens well suited for this location, and there was no history of hyphema. Infectious causes were excluded with thorough molecular and serologic testing. Sarcoidosis remained a possibility, as normal chest radiographs cannot exclude ocular sarcoid; however, the cysts (without nodules) and fibrotic membranes would be atypical for sarcoidosis and no granulomas were noted on iris biopsy. Lymphoma was excluded with flow cytometry.

This case presents a rarely described example of transition from cystic into diffuse epithelial downgrowth. Following the second PKP combined with cataract extraction and iris cyst biopsy performed at outside facility there was apparent resolution of iris cyst with subsequent development of diffuse membrane resistant to repeated Nd:YAG laser capsulotomies. In a case series by Groh et al. this transformation from cystic to diffuse is described following Nd:YAGlaser capsulotomies of the cyst. Given the recalcitrant nature of the diffuse presentation, Groh et al. emphasize the importance of block-excision technique in cases of cystic epithelial invasion with less than five clock hours of involvement [10].

A key diagnostic tool in this case was the use of argon laser photocoagulation to detect EDG. First described by Dr. Dean Rockey using a Meyer Schwickerath photocoagulator, it was noted that epithelium “fluffs up like cotton” after exposure to heat, whereas normal iris tissue will shrink [11]. This same observation can be made using Argon laser photocoagulation and is an important diagnostic procedure that can be quickly performed in the clinic. Confocal microscopy [12] and anterior segment ocular coherence tomography [13] may also bepursued to assist in diagnosis of EDG and while not pursued in this case, there may be anargument to favor these non-invasive diagnostics in the setting of preexisting inflammation. 

The use of antimetabolites for treatment of EDG was first suggested by Weiner et al. in 1989 [1]. The pyrimidine analog 5-FU works by inhibiting cell proliferation and has since been reported to successfully treat cases of epithelial downgrowth. It is, however, possible that multiple treatments are required to prevent further downgrowth and there have been reports of treatment failures after using 5-FU [14]. Successful 5-FU treatment is typically demonstrated through sustained clinical resolution, with case reports describing resolution after 5-FU treatment on external photography [15] and confocal microscopy [12]. Subsequent biopsy and repeated histopathologic evaluation is rarely performed following 5-FU treatment and to our knowledge, our case is the first to demonstrate reduction in epithelial cell burden on histopathology following 5-FU treatment. 

In conclusion, our report illustrates an unusual presentation of EDG in a young patient with granulomatous anterior and intermediate uveitis, where ocular paracentesis to rule out infection and argon laser photocoagulation proved to be relatively straightforward office-based tools that helped aid in diagnosis and management. This method of diagnosis should not be overlooked in the uveitis patient. Lastly, this case also confirms an apparent clinical and histopathologic response to 5-FU after multiple injections.

## Notes

### Patient consent

Consent to publish the case report was not obtained. This report does not contain any personal information that could lead to patient identification (in compliance with the University of Washington Human Subjects Division guidelines).

### Funding

Unrestricted departmental grant from Research to Prevent Blindness. 

### Competing interests

The authors declare that they have no competing interests.
